# Exploring the Links between Post-Industrial Landscape History and Ecology through Participatory Methods

**DOI:** 10.1371/journal.pone.0136522

**Published:** 2015-08-26

**Authors:** Kevin J. Rich, Michael Ridealgh, Sarah E. West, Steve Cinderby, Mike Ashmore

**Affiliations:** 1 Environment Department, University of York, York, United Kingdom; 2 Stockholm Environment Institute, University of York, York, United Kingdom; Institute for Natural Resource Conservation, GERMANY

## Abstract

There is increasing recognition of the importance for local biodiversity of post-mining sites, many of which lie near communities that have suffered significant social and economic deprivation as the result of mine closures. However, no studies to date have actively used the knowledge of local communities to relate the history and treatment of post-mining sites to their current ecological status. We report a study of two post-mining sites in the Yorkshire coalfield of the UK in which the local community were involved in developing site histories and assessing plant and invertebrate species composition. Site histories developed using participatory GIS revealed that the sites had a mixture of areas of spontaneous succession and technical reclamation, and identified that both planned management interventions and informal activities influenced habitat heterogeneity and ecological diversity. Two groups of informal activity were identified as being of particular importance. Firstly, there has been active protection by the community of flower-rich habitats of conservation value (e.g. calcareous grassland) and distinctive plant species (e.g. orchids) which has also provided important foraging resources for butterfly and bumblebee species. Secondly, disturbance by activities such as use of motorbikes, informal camping, and cutting of trees and shrubs for fuel, as well as planned management interventions such as spreading of brick rubble, has provided habitat for plant species of open waste ground and locally uncommon invertebrate species which require patches of bare ground. This study demonstrates the importance of informal, and often unrecorded, activities by the local community in providing diverse habitats and increased biodiversity within a post-mining site, and shows that active engagement with the local community and use of local knowledge can enhance ecological interpretation of such sites and provide a stronger basis for successful future management.

## Introduction

Extraction of coal and other minerals often involves the dumping of spoil or the stripping of surface soil. After mining has ceased, many sites receive technical reclamation, typically consisting of a cover of fertile topsoil, and sowing with productive grass/herb species or tree planting. The alternative approach is spontaneous succession, which involves no direct planting or sowing but aims only to control alien or invasive species: the typical successional communities on mine spoil are then influenced by factors such as the shallow soil depth, poor soil nutrient status and soil contamination. The rate of vegetation establishment may be slow on the infertile site conditions which remain with spontaneous succession, but the spatial heterogeneity in soil conditions can ultimately lead to a more diverse flora (e.g. [1, 2]). Unusual invertebrate communities can also develop on such sites, and studies of invertebrate groups, including ants [3] and Lepidoptera [4], suggest that naturally re-vegetated brownfield sites can support a diverse set of species. Both post-mining management and habitat heterogeneity have been identified as major factors influencing the lepidopteran and plant communities of post-mining sites [5], but the ecological benefits of retaining open, nutrient-poor habitats during restoration of post-mining sites have frequently been ignored [6].

However, the influence of local residents on the plant and insect communities that become established on post-mining sites, and their role in maintenance of a high ecological value, has rarely been considered. Furthermore, these communities, who have often suffered significant social and economic deprivation as a result of mine closures, may have important local knowledge about the history and development of a site after mining ceased. The importance of informal local knowledge to understanding the ecology and management of urban ecosystems has been established in a number of studies. For example, Tsuchiya and colleagues [7] showed the importance of interfaces between those with local ecological knowledge and those actively involved in community-based management of urban woodlands within a community in Tokyo. Andersson and colleagues [8] demonstrated the importance of informal community management practices (which are often ignored by local planners) in maintaining the ecosystem services of allotments, cemeteries and parks in Stockholm, while Barthel and colleagues [9] showed how the complex historical interactions between society and ecology have formed the environmental services provided by Stockholm’s National Urban Park, and identified the importance of local community groups and individuals in maintaining these services. However, to our knowledge, interactions between the local knowledge and actions of the local community and ecology have not been studied in the specific context of brownfield sites.

Here we describe a novel study at two post-mining sites, which involved working with local residents with the specific aims of (a) establishing restoration histories for the sites and (b) assessing links between the formal management and informal community activities and the richness of the plant and invertebrate communities now present. Our approach is a hybrid of citizen science and participatory action research (see [10] for a typology). Specifically, we engaged members of the local community through the use of Participatory GIS (PGIS). A small number of PGIS studies have used the technique to understand local knowledge of land use change and community use of a local landscape, or to access the historical aspects of cultural services [11], but, to our knowledge, they have not been applied before to assess restoration histories of post-mining sites.

The research was part of the OPAL (Open Air Laboratories) program, which aimed to engage citizens in collecting novel ecological data (e.g. [12]) and to combine this with public education and participation [13]. OPAL targeted communities in socio-economically deprived areas, such as post-mining villages, who are often under-represented in citizen science exercises [14]. The work took place in the Yorkshire coalfield, one of a number of areas in the UK in which most or all of the collieries have been closed. Many of these collieries were closely associated with local villages from which most of the workforce came. During the 1990s the potential of many of these sites for nature conservation was recognized [15]. The close proximity of the many post-mining sites to human habitation in this region, and the lack of any systematic regional program of reclamation, mean that local community activity may have had a particularly significant influence on the ecology of such sites.

## Methods

### Study location, geology and history

The two sites, Upton and Fitzwilliam, are situated south-east of the city of Wakefield. Both sites mined coal mainly from the Upper and Middle Coal Measures of the Carboniferous period. At Fitzwilliam the site is on sandstone and waste siltstones: mudstones and sandstones formed the spoil heaps that completely covered the site. The Fitzwilliam pithead was closed in 1967, but a drift mine was opened on part of the site in 1977, with final site closure in 1987. While part of the Upton site is on siltstones, mudstones and sandstones, it also has an area of Permian Magnesian Limestone. Unlike Fitzwilliam, most pit spoil from Upton was transported away from the colliery site. A disused railway cutting forms the southern boundary of the site. The Upton site closed in 1964, although clay extraction continued for the on-site brickworks. The UK Index of Multiple Deprivation shows that Upton is in the 40% most deprived areas in England, and Fitzwilliam is in the bottom 20% of areas in England, and that for both areas, deprivation levels got worse over the period 2004–2010 [16].

### Community recruitment and PGIS methods

A number of methods were used to recruit participants in order to maximize their numbers. Initially a press release appealing for volunteers to monitor wildlife on the sites and provide historical information was sent to the local newspapers. Posters were placed in local libraries, shops and post offices, and contact was made with local schools and colleges. In addition, conversations with local residents while the researchers were visiting the sites led to some individuals taking part in the study, as well as providing useful anecdotal information on site history.

Participatory GIS (PGIS) [17] workshops and on-site Rapid Appraisal GIS (RAP-GIS) were used to gather information about historical and current uses of the site (see [18] for a general description of these methods). Written consent was obtained from all participants, and the methodology was approved by the Environment Department at the University of York’s Ethics Committee. The RAP-GIS approach is particularly useful for incorporating the views of people who would not have attended a formal meeting or workshop, and was conducted at each site for a day in Spring 2011. PGIS workshops were conducted in community buildings near to each site in early 2011. Almost all of the participants had lived in the area all their lives, with most of them having had associations with the mines, either directly, or indirectly through family members. Some of the participants were members of local community groups and already took an interest in the local environment.

For the PGIS workshops, a series of size A0 topographic maps dating back over the last 40 years were provided in order to aid recollection. In addition, participants brought a selection of photographic and historical material to the workshops. At the beginning of the workshop, participants were asked a series of questions in order to create a historical timeline of what had happened at the site and when. This timeline was then used to generate maps indicating where management and development changes had occurred and the impacts these changes had on the diversity of flora, fauna, habitats and community use. This process was iterative as mapping sparked remembrance of new activities and events that were added to the timeline. Participants’ conversations were recorded to capture the details of these discussions allowing further annotation of the timeline. The workshops provided information about when and where reclamation of the sites took place, as most formal records were either destroyed or lost; indeed, at the Upton site, local residents had maps and reclamation information that would normally have been held by the local authority.

RAP-GIS involved approaching site users with an aerial photograph of the site, and asking them the following questions: which areas of the site do you use and why, which areas of the site do you not use and why, and where have you seen wildlife. They were asked to draw these areas onto the aerial photograph and notes were taken about any species they mentioned. This process provided an overview of the types of information held by a cross-section of users about the amenity value of the sites in relation to land cover, habitats and their associated biodiversity potential.

### Plant survey and habitat classification

Plant surveys using quadrats were conducted by the researchers in the grassland areas in summer 2010 (permission granted by Wakefield District Metropolitan Council). Raw data can be found in S1 Table. Sampling took place in distinct areas of grassland of potential conservation interest which were identified through discussion with local residents, as well as by using maps from site surveys conducted in 2005, and visual inspection by the researchers. Percentage abundance of flowering plants and grasses was estimated and the Domin Scale was used to quantify cover of each species. A total of 91 vascular plant species were recorded in the quadrats at Upton and 33 species at Fitzwilliam. The two sites had 27 species in common, with Upton having 64 unique species and Fitzwilliam having only 6 unique species. To supplement the quadrat surveys, a guide to positive and negative indicators of grassland condition was produced, based on a guide created for assessing road verges in limestone areas of Lincolnshire [19], for use by local volunteers. In order to assess how different areas of the two sites fitted in to the UK National Vegetation Classification (NVC) system [20], data were fed into the MAVIS (Modular Analysis of Vegetation and Interpretation System) software.

### Invertebrate data collation and habitat classifications

A species list of invertebrates was created for each site. Raw data can be found in S2 Table. Community volunteers were encouraged to submit photographs of invertebrates taken at the sites to the online species identification tool iSpot [21], and this information was complemented by data from surveys conducted by the researchers, and a small number of records from the local ecological records center. The Invertebrate Species Information System (ISIS) package [22] was used to identify broad and specific species assemblages. The assemblage codes produced by ISIS were examined for accuracy by inspecting the grid reference and the related habitat from which the record was taken. This information was used to assess if novice entomological surveyors could effectively complement traditional invertebrate recording undertaken by naturalists.

### Volunteer invertebrate surveys

Volunteers were asked to walk specified routes (transects) on a weekly basis to assess the presence and number of selected butterfly and bumblebee species, and record their observations on a form which was adapted from the Butterfly Monitoring Scheme [23] and the Bumblebee Conservation Trust Beewalk methodology [24]. Species were selected to be relatively simple to identify and not easily confused with other species. The two routes passed through woodland and grassland habitats and included areas which had been identified in discussions with community members as receiving active reclamation or having natural succession. Existing pathways were used to avoid excessive trampling of vegetation, to accommodate volunteers with low mobility, and to improve reliability and reproducibility. Volunteers were individually trained in the methodology and species identification, and surveys were undertaken in the summers of 2010 and 2011. In order to assess if volunteers were collecting reliable data in terms of identification and abundance, a group of three previously trained volunteers conducted the survey, without advice or guidance, whilst a researcher concurrently carried out an identical survey. Differences between volunteer and researcher collected data were small, with overall total differences between species agreeing within 10%.

## Results

### Community-based site history and habitat classification: Upton

**Fig 1:**
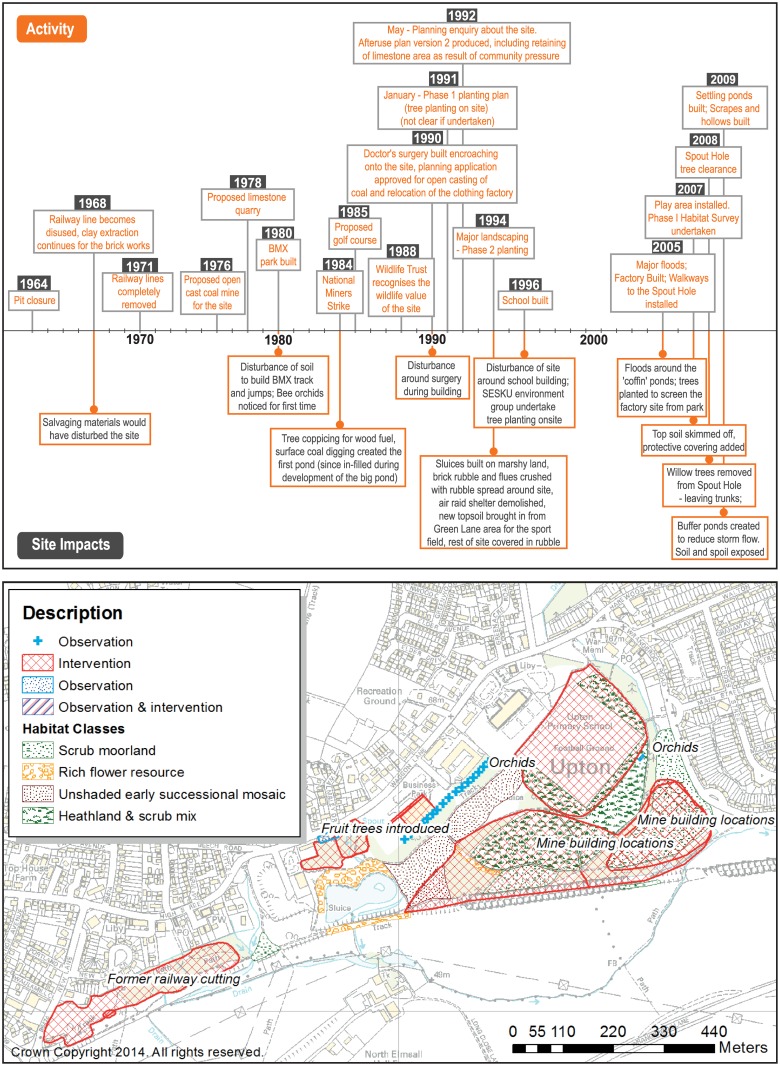
(a) timeline and (b) map of the Upton site developed using PGIS and RAP-GIS differentiating in (a) activities and site impacts and in (b) observations and active interventions. The map also shows the locations that provide high-quality invertebrate habitat classes. Use of Ordnance Survey mapping data for educational purposes licenced through the EDINA Digimap Service (see S1 Document for details of the copyright agreement).

The timeline generated from the PGIS workshop at Upton is shown in Fig 1A, which divides information into specific activities and events that had impacts on site ecology. Fig 1B shows the location of key activities (“interventions”) and impacts (“observations”) on the site identified by participants. It also identifies locations with habitats that are of high conservation value in terms of invertebrate fauna. Table 1 (Section A provides more detailed information on relevant historical activities and events in each specific area where the plant survey was undertaken, based on PGIS and other informal community engagement; it also summarizes the best fits to National Vegetation Classification (NVC) communities in each of the surveyed areas, highlighting those that have affinities with Priority Habitats within the UK Biodiversity Action Plan, and hence are of high conservation value.

**Table 1:** Summary of major NVC categories, and plants of particular interest in each area of the two sites (F = Fitzwilliam; U = Upton; UR = Upton railway cutting) alongside historic features of relevance identified by community engagement.

A: Upton			
Area	Historic and community activities	Main NVC communities	Species of interest/positive indicators
U1	Clay and brick rubble added (Technical reclamation)	Well-drained permanent pasture **Maritime cliff grassland Calcareous dune grassland**	*Festuca rubra*, *Gallium verum*, *Lotus corniculatus*, *Plantago lanceolata*, *Prunella vulgaris*
U2	Limestone area, buildings cleared, actively protected by community (e.g. site protests) (Spontaneous succession with some active management)	**Lowland limestone grassland Open habitats of spoil and scree**	*Anthyllis vulneraria*, *Campanula glomerata*, *Centaurea nigra*, *Lotus corniculatus*, *Primula veris*
U3	On geological fault line, also received active community protection (Spontaneous succession)	**Localised lowland meadow** Well-drained permanent pasture	*Lotus corniculatus*, *Rhinanthus minor*, *Sanguisorba officinalis*
U4	Recent disturbance from adding scrapes and altered site drainage (Technical reclamation)	**Open habitats of waste ground** Well-drained permanent pasture	*Campanula glomerata*, *Centaurea nigra*, *Pimpinella saxifraga* Community survey: *Campanula glomerata*, *Clinopodium vulgare*, *Hypericum perforatum*, *Silene vulgaris*
UR	Old railway cutting, lot of bike riding (Spontaneous succession)	**Lowland limestone grassland Localised lowland meadow** Well-drained permanent pasture	*Campanula glomerata*, *Centaurea nigra*, *Pimpinella saxifraga* Community survey: *Campanula glomerata*, *Clinopodium vulgare*, *Hypericum perforatum*, *Silene vulgaris*
B: Fitzwilliam			
Area	Historic and community activities	Main NVC communities	Species of interest/positive indicators
F1	Vegetation developed on old shale and spoil with little added topsoil (Technical reclamation)	**Calcareous dune grassland Open habitats of waste ground**	*Arrhenatherum elatius*, *Hypochaeris radicata* Community survey: *Rhinanthus minor*
F2	Original spoil heap area subsequently used for BMX track (Spontaneous succession)	**Maritime cliff grassland**	Community survey: *Rhinanthus minor*, *Echium vulgare*
F3	Area received a lot of brick and concrete rubble (Technical reclamation)	**Open habitats of waste ground** Well-drained permanent pasture	Community survey: *Rhinanthus minor*, orchids, *Hypericum perforatum*
F4	Area used in past by informal campers and travellers (Spontaneous succession)	**Maritime cliff grassland Lowland mesotrophic grassland Lowland limestone grassland**	*Anthyllis vulneraria*, *Lotus corniculatus*, Community survey: *Hypericum perforatum*
F5	Disturbed area affected by digging for coal and removal of trees (Spontaneous succession)	**Open habitats of waste ground Lowland mesotrophic grassland**	Community survey: *Rhinanthus minor*, orchids, *Echium vulgare*
F6	Artificially regenerated with little subsequent disturbance (Technical reclamation initially)	Well-drained permanent pasture	Community survey: *Rhinanthus minor*

The tables identify the community of best fit and other NVC communities which provided a high fit to vegetation composition in each area. Positive indicator species associated with relevant NVC communities at each site that were present in the quadrats are listed, alongside any additional positive indicators that were identified by community surveyors in the surrounding area. Note that no community surveys were reported for sites U1, U2, U3 and U4. NVC categories with affinities to Priority Habitats under the UK Biodiversity Action Plan are highlighted in bold.

The pit was closed in 1964, although some clay extraction continued after this date. No active remediation of the site took place, with natural regeneration of the spoil to form a heath and scrub matrix. Local residents noted that this provided excellent bird habitat over the following two decades. Participants highlighted that some coal remained un-extracted and plans were developed in the late 1970s to exploit the remaining resources by open-cast methods, and to quarry the limestone on the eastern half of the site. However a successful campaign by the local community during the 1980s stopped this, as they recognized and valued the biodiversity which had naturally regenerated on the site since the mine closed, and there were also concerns about the extra heavy goods vehicle traffic that the site would create in the village. Once the mine and quarry plans were abandoned, the local council developed plans for landscaping and reclamation. However, members of the local community who were keen wildlife observers protected valued species and habitats from what they perceived as destructive reclamation techniques, and negotiated a plan that preserved some of the most ecologically significant features of the site. For example, participants were aware of a variety of orchids establishing themselves by the 1980s, and described an orchid transplantation scheme that took them from areas of the site due for reclamation, to areas of the site that had naturally regenerated and would be protected. It is important to note that throughout the disputes over use and reclamation of the site, there was no organized external pressure group at work, and opposition was driven by the interests of long-term local residents. Reclamation measures that did take place included coalmine buildings being demolished and crushed for hardcore and the resulting aggregates spread over the west of the site. As part of the reclamation process in the early 1990s, a fishing pond was established; the Angler’s group formed around this have actively engaged young people with the aim of reducing anti-social activity on the site. Members of this group have also been involved in planting of ornamental cyclamen species, spindle and apple trees. Finally, in the late 2000s when a new drainage scheme was installed, local residents were involved in identifying ways to reduce its impact on biodiversity.

All the surveyed areas of the Upton site showed fits to NVC habitat classes of high conservation value (e.g. lowland limestone grassland and localized lowland meadow) or that were unexpected at such as site, including calcareous dune grassland, maritime cliff grassland and vegetation of open habitats. Table 1 also identifies the presence of a number of positive indicator species, especially of mesotrophic grassland, limestone grassland, and calcareous dune grassland. Further positive indicator species were identified by the community surveys, which were able to cover a wider area to search for relevant species, especially in the old railway cutting. Table 1 demonstrates that three of the five areas of high conservation value habitat were associated with specific community interventions or activities. The importance of community intervention to protect the Upton site is clear from the identification of lowland limestone grassland and a localized meadow community in areas U2 and U3 respectively. The disturbance caused by informal bike riding activity is likely to have contributed to the identification of these valued habitats within the railway cutting (UR). In the other two areas, site management has been the dominant factor; in U4, recent disturbance and clearance from work to alter site drainage has led to open habitat of waste ground, while in U1, the spreading of brick rubble in the early 1990s during site reclamation and landscaping created the substrate for formation of plant communities with affinities for maritime cliffs and calcareous dunes.

### Community-based site history and habitat classification: Fitzwilliam

**Fig 2:**
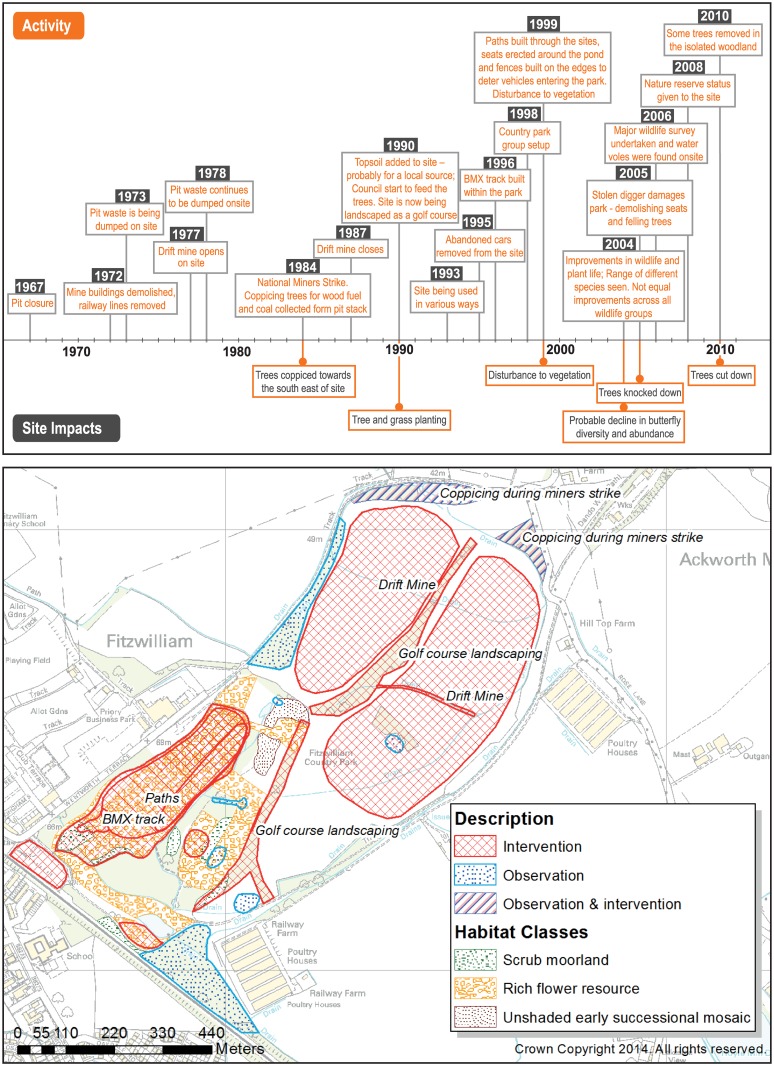
(a) timeline and (b) map of the Fitzwilliam site developed using PGIS and RAP-GIS differentiating in (a) activities and site impacts and in (b) observations and active interventions. The map also shows the locations that provide high-quality invertebrate habitat classes. Use of Ordnance Survey mapping data for educational purposes licenced through the EDINA Digimap Service (see S1 Document for details of the copyright agreement).

The timeline and map developed at the Fitzwilliam PGIS workshop, using the same format as for Upton, is shown in Fig 2, while Table 1 (Section B) summarizes the historical activities and habitat classification for the surveyed areas, as for Upton. In contrast to Upton, little spontaneous succession occurred after mine closure in 1967, as the site continued to be used to dump spoil, and in the late 1970s a drift mine was opened. Participants identified that site reclamation began in 1990, soon after the drift mine closed, with the setting out of most of the site as a golf course; landscaping used topsoil originating from nearby liquorice farms. A large scale tree planting scheme was also initiated during this early phase of the reclamation. Due to changes in council management, the reclamation was halted and the golf course plan scrapped, and by 1993 the site was mainly being used for recreation by the local residents, with a BMX track being built in 1996. Over this period, it was also noted that fly tipping and discarding of stolen vehicles were becoming prevalent at the site and a traveller’s camp occupied part of the site during the 1990s and 2000s. The effects of these activities on the ecology of the site is uncertain; however, management such as hay cropping was abandoned in these areas, which may have led to a reduction in floral species diversity. A Fitzwilliam Country Park group was set up by long-term local residents in 1998 with the aim to finish the reclamation of the site as a nature park and to reduce vandalism. They removed dumped material and improved the paths on the site. However, anti-social behavior and vandalism remain a problem at the site. Participants suggested that a reduction in butterfly diversity and abundance had taken place after reclamation.

Five of the six surveyed areas showed fits to NVC habitat classes of high conservation value or that were unexpected at such a site, including many of those identified at Upton. The community surveys at Fitzwilliam added further positive indicator species, for example, the presence of orchids and the hemi-parasite *Rhinanthus minor*. As for Upton, both historical planned management (such as vegetation control for the benefit of the anglers and to maintain public footpaths) and informal community activities were reflected in the habitat classification. However, at Fitzwilliam, areas of conservation value were associated with poorly planned reclamation and anti-social activities, rather than active community protection of areas of spontaneous succession. The outcome here was ‘wasteland’ which was left to become enriched by natural processes. The only surveyed site of low ecological value, F6, was in an area known to have been actively reclaimed for the planned golf course in the early 1990s. Areas of old spoil with little topsoil addition (site F1) and of brick rubble (site F3), from site reclamation in the 1990s, were both associated with open habitats of waste ground, reflecting the slow rate of succession on these substrates, as at Upton. The affinities with maritime cliff communities at F2 were associated particularly with the use of the area as a BMX track providing long-term disturbance. At sites F4 and F5 the disturbances that led to plant communities of high conservation value were associated with the traveller camp, and with coal digging and tree removal respectively.

### Invertebrate assemblage classification

A total of 102 invertebrate species were found at Upton, 75 of which were new records generated by this study, and 81 species were recorded at Fitzwilliam, 62 of which were new records. At both sites, the additional records provided sufficient data to calculate a range of specific and broad assemblage codes within ISIS, which were consistent with the researchers’ observations of the habitats present on the sites. Two particular assemblages of importance for invertebrate fauna were identified on the basis of this research (Table 2). Firstly, the specific assemblage type, *rich flower resource* is well represented by the invertebrate fauna at both sites, but was not accounted for by records prior to this study. The Lepidoptera and Hymenoptera groups contribute most towards this code. This code reflects and confirms that these sites, in the areas identified on Figs 1b and 2b, have excellent wildflower foraging resources. At Upton, much of this area was associated with the addition of clay and brick rubble (as at site U1); at Fitzwilliam, this was also a factor (as at site F2) while the presence of the BMX track was an additional factor (as at site F3). A total of 5% of the national invertebrate species pool associated with rich flower resource was discovered at Upton and 3.3% at Fitzwilliam. Secondly, the rarity, richness and representation parameters of the broad assemblage *unshaded early successional mosaic*, which is related to the specific assemblage type *open short sward*, all increased with addition of species identified in this study. The majority of species recorded for this particular habitat code were in areas that had most recently been disturbed, or were recorded as having plant communities of open habitats (cf. Table 1), specifically around the drainage works at Upton (as at site U4) and around the BMX track at Fitzwilliam (as at site F2).

**Table 2:** Summary of changes in site invertebrate habitat assessment for key habitat assemblages as a result of this survey.

ISIS Assemblages	Upton		Fitzwilliam	
	Pre Survey	Post Survey	Pre Survey	Post Survey
Number of species	27	102	19	81
**SAT Rich flower resource**				
Number of species	0	11	0	8
% national species pool	0	5.5	0	3.3
**SAT Open short sward**				
Number of species	2	3	1	1
% national species pool	1	1.5	0.5	0.5
**BAT** ^**a**^ **Unshaded successional mosaic**				
Species richness	3	15	2	14
Rarity (SQI)	-	100	-	107

^a^ BAT species richness is determined by the number of species associated with the broad invertebrate-habitat assemblage type. BAT Rarity is an average of species quality index for all species within the broad invertebrate-habitat assemblage type. It is calculated from the scarcity of each species on a national 10km square basis. SAT % of species pool is calculated from the number of species recorded against the total number associated with a specific invertebrate-habitat assemblage type.

### Volunteer invertebrate surveys

An insufficient quantity of survey data were provided by volunteers at Fitzwilliam to allow statistical interpretation, and therefore results are only provided for Upton (Table 3). The total number of observations for both butterflies and bumblebees were higher in the woodland rides than in the grassland, although the differences in the numbers of species were less marked. The total number of observations for both groups was higher in naturally regenerating than in reclaimed grassland. However observation numbers were lower in the naturally regenerated woodland than the reclaimed woodland, although there was variation between species. For both butterflies and bumblebees, the rarest species were only found in reclaimed areas.

**Table 3:** Summary of butterfly and bumble data from volunteer surveys at Upton.

		Reclaimed grassland	Natural grassland	Reclaimed woodland	Natural woodland
Bumblebee	Number of observations	55	78	252	200
	Number of species	6	4	7	7
Butterfly	Number of observations	39	66	121	98
	Number of species	12	10	12	12

Numbers are corrected for differences in transect length as appropriate.

## Discussion

The importance of informal local knowledge to develop an understanding of the ecology and management of urban ecosystems has been demonstrated in a number of studies [e.g. 7, 8, 9], and our study confirms this in the context of post-mining sites. The methods used to gather this local knowledge allowed us to identify areas with plant and insect communities of high conservation value within each site, and understand how the history of the site since mine closure led to particular plant and invertebrate communities developing in specific areas. The active engagement with the local community through the techniques of PGIS, in formal workshops, and RAP-GIS, through informal discussions on the sites, was critical in allowing us to develop a greater understanding of the complex factors which have led to the current patterns of plant and invertebrate diversity. In particular, understanding the spatial diversity in site management and history was crucial to interpretation of ecological patterns on the site. In addition to providing the site histories, PGIS and RAP-GIS also proved to be a valuable way of engaging volunteers in subsequent ecological surveys in areas that were likely to be of particular ecological interest, which helped to provide information on the effect of reclamation history on bee and butterfly species and on the presence of positive indicator species of conservation grasslands.

The additional invertebrate species identified in collaboration with volunteers in this study highlighted the importance of two habitats, rich flower resource and early successional mosaic, in providing a rich invertebrate fauna with unusual or rare species. The former also relates to the lowland grassland communities of conservation importance that were identified in the plant survey. Specific positive indicators of grassland quality may have value as food resources for the Lepidoptera and Hymenoptera species that contributed most to the value of this habitat. For example, *Lotus corniculatus*, an important positive indicator of grassland quality (Table 1), is also a major forage plant for important bumblebee species (e.g. *Bombus lapidarius* and *Bombus pascorum*) and butterfly species (e.g. *Polyommatus icarus*) that were identified in significant numbers in the volunteer surveys [25, 26]. The differences in bumblebee and butterfly numbers and species richness between reclaimed and naturally regenerated areas may reflect the distribution of particular plant species and habitat features which reflect the unique histories of the two sites, rather than a generalizable difference between the two forms of restoration. The evidence of active protection by members of the community of flower-rich habitats and distinctive plant species, including some transplantation, has contributed to the significance of flower-rich habitats on the sites. The new tree and shrub species that were introduced onto the Upton site by local residents, primarily for their ornamental value, may also have providing important additional foraging resources. Andersson and colleagues [8] also identified the importance of informal management activities which enhance pollinator abundance in urban environments, but this was mainly undertaken by allotment holders, who would benefit directly from these interventions.

The participatory mapping exercises also revealed that use by local residents of different areas of the site stopped or decelerated successional processes and encouraged more of the valued open mosaic habitat for invertebrates, as well as plant communities characteristic of habitats of low nutrient status with significant amounts of bare ground. These activities included tree cutting for fuel during the miner’s strike, use of motorbikes and BMX bikes on the site, and use for informal camping, as well as external planned management, such as the creation of new ponds to help downstream drainage at Upton in 2009. Regular disturbance on urban brownfield sites has been recognised as contributing to high plant and invertebrate species richness [5, 27]. Many of the locally important invertebrate species that were identified in these areas by our study require patches of bare ground; these include the attractive iridescent beetle (*Poecilus versicolor*), the pill woodlouse (*Cylisticus convexus*) the mottled grasshopper (*Myrmeleotettix maculatus*), and the yellow meadow ant (*Lasius flavus*).

The Millennium Ecosystem Assessment [28] recognised the interaction of people and landscapes in relation to land use change and management approaches. It called for a better understanding of the way society has modified landscapes and the inter-relation with ecosystem services. PGIS approaches have found some utility in mapping the cultural services afforded by different landscapes which form part of the suite of ecosystem services [29, 30], while a smaller number of mapping approaches have also tried to access the historical aspects of cultural services [11] or the local knowledge of the temporal dimension of land use change. These PGIS approaches are a way of trying to understand local knowledge of land use change and community use of a local landscape. These often involve intangible cultural services, such as sense of place, awareness of stewardship landscape responsibilities and spiritual value linked to nature that have been shown to contribute substantially to individual well-being and community social connections. In the case of the brownfield sites in our study, we have been able to identify the significance of some of these positive individual and community values in protecting and enhancing biodiversity, but also to track the long-term impact of less positive aspects such as historic socio-economic deprivation, site abandonment and a lack of long-term management strategy, in influencing the ecological and cultural services now provided by the sites.

Our research demonstrated that our understanding of the ecology of brownfield sites may require the gathering of detailed informal knowledge of the interactions between the site and the local community over time. This is because the current ecological value of such sites is strongly influenced by time since abandonment, and informal management activities such as those described here can have a significant impact over that time period. Many brownfield sites will have had complex histories since their abandonment, which, as for our sites, are undocumented or only partially documented, and interpretation of current ecological patterns and spatial diversity may depend on such information. Despite the fact that the conservation value of brownfield sites in general, and post-mining sites in particular, is increasingly recognised, urban and industrial areas have traditionally been neglected in ecological survey programmes. For example, in the UK, Open Mosaic Habitat on Previously Developed Land was only identified in 2007 as a new Priority Habitat within the National Biodiversity Action Plan [31], and there is simply not enough data available to assess what proportion of the UK’s thousands of brownfield sites would meet the ecological criteria for this Priority Habitat [32]. Hence there is an important potential role for engagement of local communities in improving knowledge and understanding of such sites.

Although engagement of communities in ecological surveys has become more widespread, to our knowledge no study has adopted the approach described here, where active engagement with the local community provided an enhanced understanding of how site history has influenced the ecology of different areas. Many features of our findings will be unique to these sites or to the Yorkshire coalfield region, and using local history as a ‘hook’ for engagement may have been particularly effective here because of the emotive cultural impact of the large scale closure of mines, and accompanying loss of thousands of jobs in the Yorkshire region. Nevertheless, the approach that we have taken, and in particular the use of PGIS and RAP-GIS methods to stimulate discussion and focus field investigations, may be useful to inform future practice in community engagement with the ecology of brownfield sites or of other areas that have experienced rapid land use change which has not been well documented.

## Supporting Information

S1 Document Extracts of the End User Licence Agreement for use of Ordnance Survey map data. (DOCX)

S1 Table Plant quadrat data for the study sites (Upton and Fitzwilliam). (XLSX)

S2 Table Invertebrate data for the study sites (Upton and Fitzwilliam). (XLSX)
