# Supplementary material for: Exploring the Links between Post-Industrial Landscape History and Ecology through Participatory Methods
Source: PLoS One. 2015 Aug 26;10(8):e0136522. doi: 10.1371/journal.pone.0136522 (PMC4550255; doi:10.1371/journal.pone.0136522)
Supplement: S1 Document — (DOCX) [file pone.0136522.s001.docx]

The following are excerpts from the End User Licence Agreement between the University of York as an EDINA Digimap user and Ordnance Survey, which permit us to publish mapping images.

“End User Licence Agreement

IMPORTANT NOTICE:

This end user licence agreement (Licence) is a legal agreement between you and the Secretary of State for Business, Innovation and Skills acting through Ordnance Survey, (whose principal place of business is at Explorer House, Adanac Drive, SOUTHAMPTON, UK, SO16 0AS) (referred to as Ordnance Survey, us, our or we in this Licence).

It is a Licence for the use of Ordnance Survey mapping data through the EDINA Digimap Service for educational purposes.”

The relevant sections of this licence are we may:

“3.3.8 publish copies of your Academic Works and Research Works in academic journals, periodicals and other publications, for the purpose of communicating the results of your scholarly work:

a) in printed form; or

b) in electronic form, provided that the mapping images included in such publication comply with the requirements of sub-clause 5.1.4;

5.1.4 ensure that any mapping images that are included in Academic Works, Presentation Materials, Research Works and Teaching Materials, which are, or are likely to be, distributed or otherwise made available to persons who are not Authorised Users (including but without limitation by way of publication on the world wide web), shall:

a) be in a Raster Format only;

b) not be geo-referenced;

c) be no larger (in scale, coverage area, number of features) than is necessary to fulfil the purpose for which the map is being used;

d) include additional information on/alongside/with the map which facilitates the purpose for which the map is being used; and

e) you shall take adequate and technological measures to prevent third parties from being able to access, use and/or extract any Ordnance Survey Licensed Data from such materials/media.”
